# Recognition of Food Processing and Its Association with Consumption Patterns and Nutrient Intake Among Saudi University Students

**DOI:** 10.3390/foods15172955

**Published:** 2026-08-22

**Authors:** Wajd D. Alomari, Noha M. Almoraie

**Affiliations:** Department of Food and Nutrition, Faculty of Human Sciences and Design, King Abdulaziz University, Jeddah 3270, Saudi Arabia; wmusaalomari@stu.kau.edu.sa

**Keywords:** ultra-processed foods, food processing, health habits, NOVA classification, consumer awareness

## Abstract

Previously, food processing and nutrition sciences operated as distinct scientific domains; however, increasing health concerns have highlighted the need for integrated perspectives and practical food classifications. This study aimed to assess participants’ knowledge of food processing classification according to the NOVA system and investigate its association with ultra-processed food (UPF) consumption patterns and nutrient intake. A cross-sectional study of 403 healthy students (18–30 years) was conducted. Participants were asked to classify each food according to its level of processing. Dietary intake was assessed in a subsample of 190 participants who completed the 24-h dietary recall assessment and had complete dietary data available. Higher food processing knowledge was associated with lower UPF consumption (B = −0.241, 95% CI: −0.414 to −0.068; *p* = 0.006). Participants with lower knowledge exhibited higher mean consumption of bread and bakery products, breakfast cereals, savory snacks, dairy products, ready-to-eat meals, and processed meats. After adjustment for age, no significant differences in nutrient intake were observed between knowledge groups.

## 1. Introduction

The food industry plays a vital role in providing safe, convenient, and shelf-stable food items to consumers globally. However, food processing and its relationship with health are significant concerns due to the impact of this processing on the nutritional profiles of various foods [1]. Food processing is instrumental in modern society, influencing dietary consumption as well as economic conditions, public health, and environmental sustainability [2]. Throughout history, food processing and technology have greatly impacted the availability and composition of food. However, there is a growing need to simultaneously consider the health potential and environmental footprint of processed food [3]. Several systems of classification have been developed to elucidate the different forms of food processing. Among the most widely applied in nutrition research are those proposed by NOVA, the International Agency for Research on Cancer, the International Food Information Council, and the University of North Carolina [4]. While some systems classify foods according to the extent and type of processing, others emphasize differences in formulation and ingredient composition. Monteiro et al. [5] suggested the existence of three groups of processed foods and argued that food processing was being overlooked in research and policy [5]. Their work laid the foundation for the NOVA classification, which, according to a systematic review, is widely used and introduced the term ultra-processed foods (UPFs) [6]. Subsequent publications reported refined data and expanded the classification by dividing the third category into ‘processed food’ and ‘ultra-processed food’ categories. Thus, NOVA classifies all foods and food products into four groups: (1) unprocessed or minimally processed foods; (2) processed culinary ingredients; (3) processed foods; and (4) UPFs [5]. 

Growing evidence indicates that high consumption of UPFs is associated with poorer diet quality and adverse health outcomes. Diets rich in UPFs are generally characterized by excessive intakes of energy, added sugars, saturated fat, and sodium, together with inadequate consumption of fruits, vegetables, whole grains, and other nutrient-dense foods [7,8]. Furthermore, systematic reviews and meta-analyses have consistently reported associations between higher UPF consumption and an increased risk of obesity, type 2 diabetes, hypertension, dyslipidemia, cardiovascular disease, and all-cause mortality [9,10,11]. These findings have highlighted the importance of strategies aimed at reducing UPF consumption and promoting healthier dietary choices. Reducing the consumption of ultra-processed foods requires consumers to correctly identify foods according to their degree of processing [12,13]. Although the terms food processing knowledge, awareness, literacy, and recognition are frequently used in the literature, they represent distinct concepts. Food processing knowledge refers to an individual’s understanding of food processing concepts and classification systems [13,14], whereas food processing awareness reflects familiarity with the health implications of processed foods. Food processing literacy encompasses the ability to obtain, interpret, and apply processing-related information when making food choices [14]. In the present study, food processing recognition refers to the ability to correctly identify the level of processing of food products according to the NOVA classification and represents the construct evaluated throughout the study.

Previous studies have shown that consumers often experience difficulty distinguishing foods according to their degree of processing, particularly when products have similar appearances despite substantial differences in formulation or processing [12,13,15]. Misclassification of foods may reduce consumers’ ability to make informed dietary choices and may contribute to the unintentional consumption of ultra-processed foods. However, most previous studies have focused on describing recognition accuracy or examining the health effects of UPF consumption [12,13,15], whereas limited evidence is available regarding whether food processing recognition is associated with actual food consumption patterns and nutrient intake, particularly among Middle Eastern populations. The present study is a secondary analysis of the same participant cohort used in our previous publication [16]. However, the previous study investigated the association between ultra-processed food consumption and obesity indicators, Mediterranean diet adherence, and nutrient intake, whereas the current study addresses a different research question by examining food processing recognition and its association with food consumption patterns and nutrient intake. Therefore, this study aimed to assess recognition of food processing and examine its association with food consumption patterns and nutrient intake among Saudi university students. Based on the available evidence, we hypothesized that (H1) participants with higher food processing recognition would exhibit healthier food consumption patterns than those with lower recognition, and (H2) participants with higher food processing recognition would have a more favorable nutrient intake profile.

## 2. Materials and Methods

### 2.1. Study Design and Participants

This study employed a cross-sectional, quantitative, and descriptive design. It aimed to explore the association between participants’ knowledge of processed foods based on the NOVA classification, their intake of these foods, and how their knowledge of UPFs influences their diet quality. University students were selected as the study population because they represent a young adult population with increasing autonomy in food selection and purchasing [17,18]. In addition, university-based electronic communication provided feasible access to this population and facilitated participant recruitment and completion of the study procedures. This population was therefore considered appropriate for examining food-processing classification knowledge and its relationship with dietary patterns. The participants were requested to provide assessments regarding the degree of processing and consumption of various food products. A minimum sample size of 383 participants was required for the knowledge assessment based on an a priori sample size calculation using the total student population at King Abdulaziz University (approximately 75,727 students), a 95% confidence level, a 5% margin of error, and a 50% response distribution [19]. Accordingly, 403 participants were included, comprising 182 males (45.2%) and 221 females (54.8%), with a mean age of 21.78 ± 2.73 years. Participants were aged 18–30 years and completed the first part of the study, which assessed knowledge of food processing classification, recognition of the processing level of selected food products, and frequency of consumption of 16 selected food products. All 403 participants were invited to voluntarily participate in the dietary assessment. After applying the prespecified eligibility criteria, 190 participants with complete dietary and anthropometric data were included in the second part of the study. This subsample comprised 94 males (49.5%) and 96 females (50.5%), with a mean age of 20.98 ± 2.38 years. Dietary intake was assessed using two non-consecutive 24 h dietary recalls to quantify UPF consumption and examine its association with obesity indicators and nutrient intake. Baseline characteristics were generally comparable between the 190 dietary assessment participants and the 213 nonparticipants, with no statistically significant differences observed across the assessed characteristics, except for marital status (Supplementary Table S1). Data were collected between February 2023 and June 2024. The recruitment and survey distribution procedures were consistent with those described in our previous study [16]. We excluded individuals who were previously diagnosed with chronic disease (e.g., eating disorders, diabetes, cancer, or cardiovascular disease), as well as pregnant and breastfeeding women. The Ethics Committee of Human Research of the Faculty of Medicine of King Abdulaziz University, Jeddah, Saudi Arabia, approved this study (Reference No 25-23). Written informed consent was obtained from all participants, who were also informed of their right to accept or decline participation and to withdraw at any time without providing a reason.

### 2.2. Food-Processing Knowledge Assessment

Prior to the main study, to ensure content validity, the questionnaire was reviewed by experts in the department. A pilot study involving 10 participants was conducted before full distribution. Internal consistency reliability was evaluated using Cronbach’s alpha, yielding a value of 0.827 for the knowledge scale, indicating acceptable reliability. The pilot study was conducted to evaluate the clarity, comprehensibility, content, and layout of the questionnaire, as well as the feasibility of the data collection procedures and the time required for completion. Participants provided feedback on the wording and usability of the questionnaire, and minor revisions were made accordingly before the final version was administered. 

An invitation to participate was distributed through the university’s email system via the Deanship of Graduate Studies. The purpose of the study was explained clearly, and participants were guaranteed confidentiality and anonymity.

Knowledge and consumption of foods according to their degree of processing were assessed using a researcher-developed questionnaire based on the NOVA food classification system. The questionnaire included 16 food items commonly consumed by young adults, comprising eight minimally processed foods (grilled chicken, eggs, white rice, banana, dates, lettuce, black coffee, and tea) and eight ultra-processed foods (packaged white bread, chicken burger, beef burger (commercially prepared burgers), chocolate bars, cookies, soft drinks, energy drinks, and packaged French fries). The questionnaire was designed to evaluate participants’ recognition of selected food products representing minimally processed and ultra-processed foods. Therefore, it was not intended to assess recognition across all four NOVA food groups.

Food items were selected according to three predefined criteria: (1) classification within the NOVA framework; (2) their frequent consumption among Saudi young adults, as reported in previous local dietary studies; and (3) representation of major food categories commonly consumed by this population, including beverages, grain products, snacks, sweets, and ready-to-eat foods [20,21]. The final selection was intended to include foods that were both familiar to participants and representative of varying levels of food processing. For each food item, participants were asked to identify its level of processing by selecting one of the following categories: minimally processed food, processed food, ultra-processed food, or “don’t know”. 

The knowledge score was calculated by summing the number of correctly classified items, with higher scores indicating greater knowledge of food processing classifications. Cronbach’s alpha value for assessing the internal consistency reliability of the knowledge sections was 0.827. Participants were also asked to report their usual consumption frequency of each food item during the previous month using a semi-quantitative food frequency scale ranging from “never or less than once per month” to “6+ times per day.” The reported consumption frequencies were converted into a continuous measure of servings/day using the median value within each frequency category. The categories “never or less than once per month,” “1–3 times per month,” “once per week,” “2–4 times per week,” “5–6 times per week,” “once per day,” “2–3 times per day,” “4–5 times per day,” and “6 or more times per day” were converted to 0, 0.07, 0.14, 0.43, 0.79, 1, 2.5, 4.5, and 6 servings/day, respectively. A medium portion size was assumed for all food items. Separate consumption scores were calculated for minimally processed foods and ultra-processed foods by summing the frequency scores of the corresponding items. Higher scores reflected greater consumption of foods within each processing category.

### 2.3. Study Questionnaire

Data were collected using a self-administered online questionnaire distributed to participants via an email link. The questionnaire was developed using Google Forms; it consisted of closed-ended questions written in Arabic. The online survey clearly outlined the inclusion criteria for the study on the first page. Furthermore, the initial page of the questionnaire provided details regarding the study’s objectives and target population. The questionnaire also explicitly stated that participation in the study was voluntary and confidential. Before data collection, all participants were required to provide online consent by signing a consent form. Anonymity was ensured, and no sensitive information was collected. The participants were free to discontinue participation at any point if they felt uncomfortable. 

The questionnaire comprised four sections. The first section focused on the sociodemographic characteristics of the participants, including sex, age (in years, continuous), marital status (single, married, or divorced/widowed), living condition (with family or alone), and monthly family income in Saudi Riyals. The second section investigated the dietary and health habits of the participants, with a particular focus on identifying lifestyle choices, such as smoking or using other forms of tobacco (yes or no); physical activity (active or inactive); dietary habits such as fruit and vegetable intake (yes or no) and the frequency of consumption of fast foods and soft drinks (once/day, 5–6 times/week, 2–4 times/week, once/week, or never); sleep hours per night during weekdays; and daily screen time (TV, videos, internet, social media) on weekdays. 

The third section assessed the participants’ understanding of UPFs, using three questions originally used in the study by Bolhuis et al. [22]. The first question was, ‘Do you know what UPFs are?’ with response options of ‘Yes’, ‘No’, or ‘Maybe’. The second question was, ‘According to you, UPFs are…?’ and allowed for multiple selections from the following options: ‘Foods composed of more than five ingredients’, ‘food products subjected to a series of industrial processing’, ‘genetically modified products’, ‘food products that contain artificial ingredients’, or ‘I don’t know what ultra-processed foods are’. The third question was, ‘What do you think is the best definition of UPF?’ with the following response choices: ‘Foods with many processes performed by the food industry’ or ‘Foods with many ingredients in their formulations’. The fourth section aimed to identify the assumed level of food processing for each item, along with its frequency of consumption, using images. The questionnaire consisted of closed-ended questions for 16 food items. Consumers visualized pictures of each food item, classified the degree of processing, and indicated the frequency of consumption. Participants’ identification of food processing type was categorized into two groups: low knowledge (0–8) and high knowledge (9–16). To determine the consumption of each item, the participants could choose from nine options: never or less than once a month, 1–3 times per month, once a week, 2–4 times per week, 5–6 times per week, once a day, 2–3 times per day, 4–5 times per day, and 6+ times per day. Each food item’s medium serving size and picture were extracted from the validated Myfood24 website “https://myfood24.org/en/login (accessed on 12 November 2023)” [23,24].

### 2.4. Dietary Assessment

All participants were invited to voluntarily participate in a follow-up involving two non-consecutive 24 h dietary recalls. Subsequently, the final analysis included 190 participants (94 males and 96 females). Participants received written instructions to record all foods and beverages they had consumed at home and outside during the previous 24 h on two non-consecutive days (one weekday and one weekend). Each page of the instructions provided sections to log the quantity, time, occasion, brand name, and food source of their foods and beverages. Afterwards, recalls were reviewed for exclusions or errors. The details of the dietary assessment were described in our previously published study [16].

### 2.5. Statistical Analysis

Analyses were performed using IBM SPSS Statistics for Windows, version 29 (IBM Corp., Armonk, NY, USA). Descriptive statistics included frequencies and percentages for categorical variables and means with standard deviations (SDs) and standard errors (SEs) for continuous variables. Categorical variables are presented as frequencies with corresponding percentages. This analysis was based on two aspects. We explored the participants’ understanding of the classification of 16 food items based on the NOVA system, which categorizes foods based on the level and purpose of industrial processing. Participant characteristics, including demographics, physical activity, smoking status, and fruit and vegetable intake, were then assessed in relation to their knowledge of industrial processing. The total score ranged from 0 to 16 and was analyzed as a continuous variable in the principal regression analysis. For group comparisons, knowledge was categorized into low (0–8) and high (9–16) based on the midpoint of the total possible score range. To determine the association between participants’ food consumption and their ability to classify the level of food processing in relation to demographic characteristics and lifestyle factors, the chi-squared (χ^2^) test and Fisher’s exact test were performed for categorical variables and unadjusted linear regression models were used for continuous variables. Adjusted linear regression analyses were used to assess the relationship between consumption of UPFs and knowledge of processing classification. This study fitted two models: Model 1 was adjusted for sex, age, academic level, income, and living arrangement, while Model 2 included additional adjustments for level of physical activity, smoking status, fruit and vegetable intake, and sleep duration. Statistical significance was defined as a *p*-value < 0.05.

## 3. Results

### 3.1. Assessment of Understanding of UPF

Of the 403 participants, only 58 knew the meaning of UPF. In all, 110 were familiar with the term, but more than half (235 of 403) were unfamiliar. Most participants identified UPFs as foods that underwent industrial processing and contained artificial ingredients. When asked to define UPFs in a multiple-choice question, the majority of participants identified them as ‘food products that undergo extensive industrial processing’ (54.8%) or ‘products containing artificial ingredients’ (38.7%). Similarly, in response to a follow-up question on the most accurate definition of UPFs, 75.2% of respondents characterized UPFs as ‘foods subjected to multiple industrial manufacturing processes’ (Table 1).

**Figure 1:**
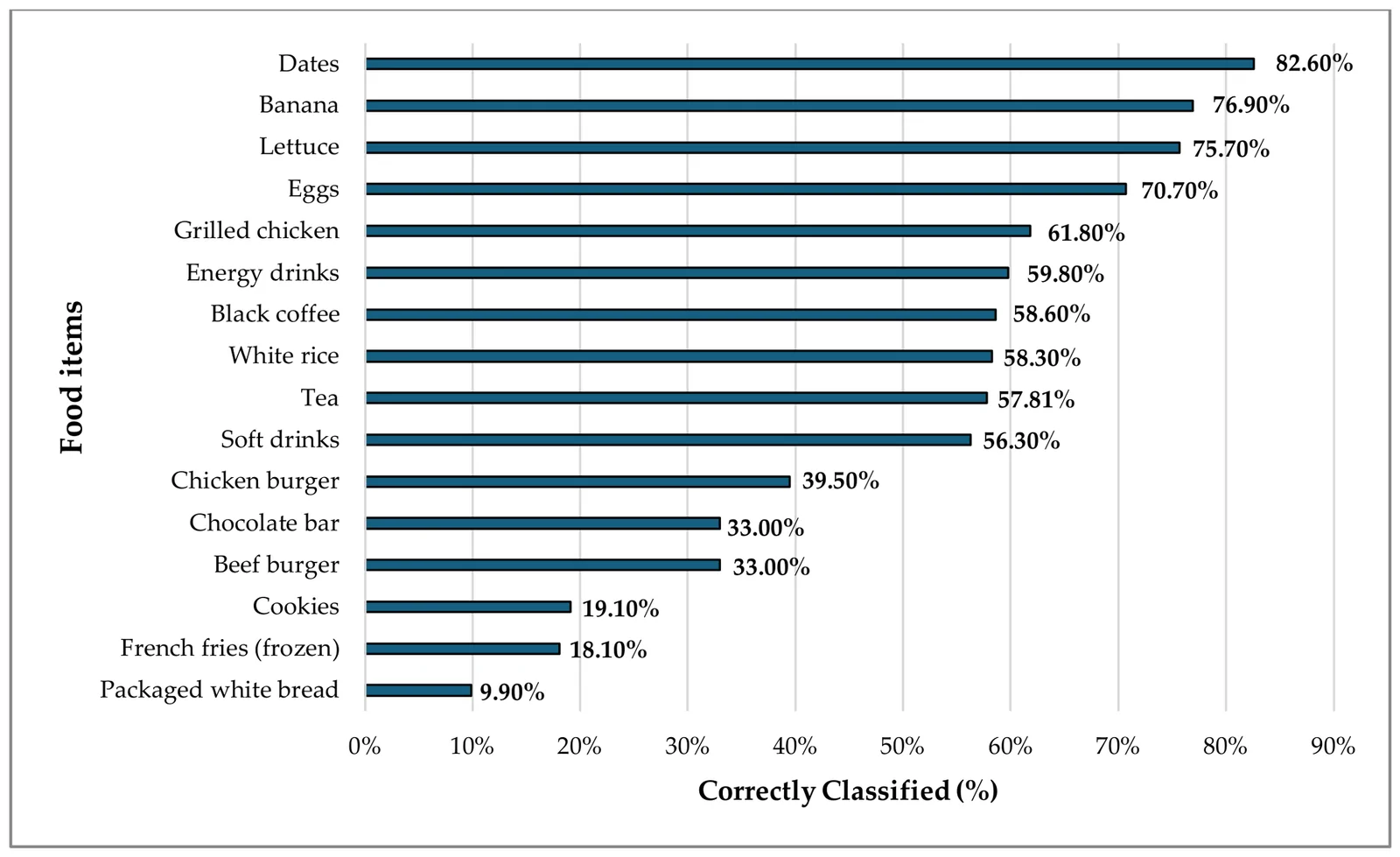
Participants’ classification of various food items according to their processing level (*n* = 403).

The recognition rates for the 16 food items, including minimally processed foods and UPFs, are shown in Figure 1. The ultra-processed items were the most challenging to distinguish among various categories. Notably, packaged white bread had the lowest rate of accurate identification, with 90% of the participants failing to recognize it as a UPF, followed by French fries, cookies, chocolate bars, beef burgers, and chicken burgers. Conversely, dates, bananas, lettuce, eggs, and grilled chicken were the most correctly identified food items.

### 3.2. Food Processing Knowledge by Participants’ Characteristics

Table 2 shows the relationship between the knowledge of processed food classification and the sociodemographic and health-related variables of the 403 participants. Participants with high knowledge were significantly older on average (22.05 ± 2.91 years) than those with low knowledge (21.47 ± 2.47 years). Although the majority of participants were single across both groups, a higher proportion of married individuals had high knowledge of UPFs (7.9%) compared to low knowledge (1.1%).

### 3.3. Consuming UPFs and Knowledge of Food Processing Classification

Higher knowledge of food processing classification was significantly associated with lower UPF consumption (Table 3). In the fully adjusted model, each one-point increase in the knowledge score (0–16) was associated with a 0.241-unit decrease in the UPF consumption score (B = −0.241, SE = 0.088, 95% CI: −0.414 to −0.068; *p* = 0.006). The association remained statistically significant after adjustment for age, gender, marital status, living arrangements, monthly family income, smoking, sleep duration, fruit and vegetable consumption, and physical activity. A similar inverse association was observed in the crude model (B = −0.258, SE = 0.086, 95% CI: −0.428 to −0.089; *p* = 0.003) and Model 1 (B = −0.257, SE = 0.087, 95% CI: −0.428 to −0.087; *p* = 0.003), indicating that the association was relatively consistent across the different levels of adjustment.

### 3.4. Food Processing Classification Knowledge, Dietary Patterns and Nutrient Intake

Table 4 presents the distribution of total daily energy intake from UPFs among 190 participants. In total, participants consumed an average of 978.37 kcal/day from UPFs, with bread/bakery products being the highest contributor (162.83 ± 11.33 kcal), followed by ready-to-eat/heat meals (154.22 ± 13.17 kcal) and processed meat (88.47 ± 10.86 kcal). Individuals with lower knowledge of processed food classification had higher mean UPF intakes of bread and bakery products, breakfast cereals, snacks and savory items, dairy products, ready-to-eat or ready-to-heat meals, and processed meat compared with participants with higher knowledge. 

Table 5 presents participants’ nutrient intake according to their levels of knowledge of processed food classification. Individuals with lower knowledge reported higher mean intakes of fats, trans fats, and saturated fats compared with those with higher knowledge. Conversely, participants with higher knowledge had a higher mean fiber intake than those with lower knowledge. After adjustment for age, some descriptive differences were observed between the knowledge groups; however, none were statistically significant, as all 95% confidence intervals included the null value.

## 4. Discussion

The prominent role of UPFs in current food environments, together with the expanding body of evidence linking their consumption to adverse health consequences, has led to growing support for regulatory approaches such as front-of-package labeling systems that enable consumers to more readily recognize and evaluate these products before purchase [25]. This study highlights the importance of individuals’ knowledge of food processing classification in relation to demographic characteristics, UPF consumption, nutrient intake, and UPF dietary patterns. The findings revealed three key observations. First, participants demonstrated substantial difficulty in correctly identifying several UPFs, particularly packaged white bread, French fries, cookies, chocolate bars, and commercially prepared burgers. Second, lower knowledge of the extent of industrial food processing was associated with higher consumption of UPFs, even after adjustment for potential confounders.

One of the most notable findings was the limited ability of participants to correctly classify several commonly consumed UPFs. While unprocessed foods such as dates, bananas, lettuce, eggs, and grilled chicken were readily identified, participants frequently misclassified packaged white bread and other industrially manufactured foods. In particular, nine out of ten participants failed to recognize packaged white bread as an ultra-processed product. This finding is important because foods such as commercially packaged bread are often perceived as staple foods rather than industrial formulations, despite meeting the NOVA criteria for ultra-processing. Similar observations have been reported in previous studies, showing that consumers can easily identify highly visible UPFs such as soft drinks and confectionery products but experience greater difficulty when classifying foods that occupy a more ambiguous position within the food supply [26]. A qualitative study by Barrett et al. [27] highlighted that many participants perceived public education campaigns as essential complements to food labeling strategies, emphasizing that consumers require adequate knowledge to understand and interpret the term “ultra-processed foods.” Furthermore, evidence from studies supports this view, as exposure to mass media campaigns was associated with greater awareness of the term “ultra-processed products” compared with individuals who had not encountered such campaigns [28,29].

Research has found a correlation between the intake of UPFs and young age [30,31]. This study focused explicitly on young adult students, who tend to follow unhealthy habits and have a higher chance of consuming UPFs than older individuals. A factor influencing students’ dietary habits is the global ‘Westernization’ of diets, a trend observed worldwide. This phenomenon involves forsaking traditional dietary customs in favor of increased consumption of sugar, sweets, sweetened beverages, fast food, red meat, and processed foods [32,33,34]. The Western diet, characterized by a high intake of fast food and sweetened drinks and reduced consumption of traditional foods, has contributed to a decline in diet quality, highlighting the relationship between changing dietary patterns and their consequences. Several studies have identified a correlation between lifestyle patterns and Western culture, linking the consumption of high-energy foods to unhealthy lifestyles [35]. This poses a threat to the health of young adults [36].

Dietary habits are a pivotal aspect of lifestyle, indicating the need for lifestyle interventions to support healthy food choices and eating behaviors. These habits include consuming fast foods, soft drinks, and UPFs, accompanied by inadequate amounts of fruit and vegetables [16,37]. Similarly, our previous study demonstrated that higher UPF consumption was associated with greater intakes of total fat, sugar, and energy [16]. Consistent with these findings, the present study found that higher knowledge of food processing classification was associated with lower UPF consumption and a more favorable nutrient profile in the present study, these findings should be interpreted within the context of ongoing debate surrounding the NOVA classification system. Emerging evidence suggests that the health effects of UPFs may vary across food subgroups, with some products, such as sugar-sweetened beverages and processed meats, consistently associated with adverse health outcomes, whereas certain fortified grain products and breakfast cereals may contribute beneficial nutrients to the diet [38,39]. Therefore, food processing classification should not be considered in isolation from nutritional composition. Nonetheless, the UPF categories most frequently consumed by participants with lower knowledge in the present study, including processed meats, ready-to-eat meals, savory snacks, and bakery products, are generally characterized by less favorable nutrient profiles, supporting the relevance of improving knowledge of food processing as one component of broader nutrition education strategies. However, the observed association should be interpreted with caution, as individuals with greater knowledge of food processing may also be more health-conscious or have greater exposure to nutrition education, factors that could independently influence dietary choices.

Barrett et al. [27] highlighted that while front-of-package labeling (FoPL) may improve consumers’ ability to identify UPFs, its effectiveness depends on clear communication and public understanding. Participants emphasized the importance of complementary education campaigns to improve knowledge of UPFs and support informed food choices. The study also identified concerns regarding conflicting messages between UPF labels and existing health rating systems, as well as the potential oversimplification of food healthfulness when relying solely on processing level. Consequently, the authors suggested that integrating UPF classification into existing nutrient-based labeling systems may provide a more balanced and transparent approach to guiding consumer decisions. Furthermore, they concluded that labeling strategies should be accompanied by broader policies addressing the affordability, accessibility, and marketing of healthier foods to achieve meaningful improvements in population diets [27].

Notably, our study found that individuals with high UPF consumption not only had lower recognition rates for these foods and a higher likelihood of soft drink and fast-food intake but also engaged in less physical activity and consumed fewer fruits and vegetables. Correspondingly, a study conducted in Spain revealed that individuals who consumed little-to-no UPFs were 17% better at identifying UPFs, whereas frequent consumers of UPFs predominantly fell in the category of low UPF identification (97.9%) [40]. Moreover, individuals who better identified UPFs were more likely to be women aged 21 years or older, consume at least two servings of fruit daily, maintain a regular exercise routine, live independently, and be familiar with UPFs [40]. Research has identified several factors that encourage healthy eating behaviors among young adults, including a deeper understanding of nutrition, receiving education on food and meal planning, being dedicated to food preparation, and participating in physical activity, which were all critical drivers of healthy eating habits; moreover, these factors serve as effective remedies to overcome obstacles hindering young adults from adopting healthy diets [41,42]. Notably, Spronk et al. [41] conducted a systematic review of 29 studies that provided essential insights into knowledge and food intake in adults aged 18 years and above. These studies revealed that enhanced nutritional knowledge was linked to a positive association with increased consumption of vegetables and fruits and reduced fat intake. Furthermore, greater nutritional knowledge was associated with an increased intake of cereals or fish, decreased consumption of sugary beverages, and increased intake of fiber, calcium, and essential food groups that conformed to public health recommendations [43].

Some researchers have argued that public health recommendations should consider both the degree of processing and the nutritional quality of foods rather than relying solely on processing classifications [38,39]. Ludwig and colleagues similarly emphasized that certain UPFs may play a beneficial role in supporting nutrient adequacy and food accessibility, particularly among socioeconomically disadvantaged populations [44]. Therefore, while reducing the consumption of nutrient-poor UPFs, such as sugar-sweetened beverages, confectionery products, and highly processed fast foods, remains an important public health goal, caution is warranted when interpreting all UPFs as equally detrimental. Future research should continue to examine individual UPF subgroups to better understand how food processing, nutrient composition, and dietary patterns jointly influence health outcomes. 

This study has some limitations. Owing to the cross-sectional nature of the data, temporal causality could not be proven, and the possibility of reverse causation cannot be ignored. Therefore, it cannot be determined whether greater knowledge leads to healthier food choices or whether individuals with healthier diets are more likely to acquire nutrition-related knowledge. Furthermore, participants classified foods according to their own perception of processing level, which may not always correspond to the actual formulation or manufacturing process of the products. Because processing characteristics are not always apparent from product appearance or labeling, some degree of misclassification may have occurred, potentially introducing information bias. Nevertheless, this reflects consumers’ real-world recognition of food processing, which was the primary focus of the study. Second, dietary intake was assessed using self-reported methods, including a food frequency questionnaire and 24-h dietary recall, which are subject to recall bias and potential misreporting. Third, the food processing classification questionnaire was researcher-developed and, although it was based on the NOVA classification framework, has not undergone formal validation. In addition, the study population consisted of young adults from a single university setting, which may limit the generalizability of the findings to other age groups or populations with different socioeconomic and cultural backgrounds. Finally, although several potential confounders were considered in the analyses, residual confounding from unmeasured factors such as nutrition education, food purchasing behaviors, and health consciousness cannot be excluded. 

Despite these limitations, this study has several strengths, including the relatively large sample size, the assessment of both knowledge and consumption of foods according to their level of processing, and the use of both food frequency questionnaire and 24-h recall data to provide complementary information on dietary patterns and nutrient intake. Furthermore, this study contributes novel evidence regarding the relationships between food processing classification knowledge, ultra-processed food consumption, and nutrient intake among young adults. Future research should explore context-specific strategies for improving food processing classification knowledge among Saudi university students, taking into account existing national nutrition initiatives and university-based health promotion programs.

## 5. Conclusions

In conclusion, knowledge of food processing may play a role in dietary choices among Saudi university students. Our study highlights a significant knowledge gap regarding the identification of UPFs, particularly products such as packaged white bread, French fries, cookies, and commercially prepared burgers. Participants with greater knowledge of food processing classification were more likely to report lower consumption of UPFs. These findings suggest a potential association between understanding food processing and dietary choices. Given the widespread consumption of UPFs among Saudi university students, strategies to improve knowledge of food processing classifications may warrant further investigation. However, the effectiveness of nutrition education, food labeling initiatives, or other interventions was not evaluated in the present study. Further longitudinal and experimental studies are needed to determine whether improving food processing classification knowledge leads to sustained changes in dietary behaviors and long-term improvements in dietary quality and health.

## Figures and Tables

**Table 1:** Participants’ knowledge and understanding of the concept of ultra-processed foods (*n* = 403).

Knowledge and Understanding of the Concept of Ultra-Processed Food (Three Questions)	*n* (%)
**Q1: Do you know what ultra-processed foods are?**	
Yes	58 (14.4)
No	235 (58.3)
Maybe	110 (27.3)
**Q2: According to you, ultra-processed foods are (multiple choices): (1)**	
Foods composed of more than five ingredients	55 (13.6)
Food products that undergo extensive industrial processing	221 (54.8)
Genetically modified products	122 (30.3)
Food products that contain artificial ingredients	156 (38.7)
I don’t know what ultra-processed foods are	118 (29.3)
**Q3: What do you think is the best definition of UPF?**	
Foods subjected to multiple industrial manufacturing processes	303 (75.2)
Foods with many ingredients in their formulation	100 (24.8)

(1) Category adds up to more than 100% as participants could select more than one option.

**Table 2:** Association between participants’ knowledge of food processing classification and demographic and lifestyle characteristics (*n* = 403).

Characteristics	Ultra-Processed Food Classification Knowledge Using NOVA			
	Total	Low Knowledge (1)	High Knowledge	*p*-Value (2)
	*n*= 403 *n* (%)	*n*= 188 *n* (%)	*n*= 215 *n* (%)	
**Age (years) (Mean ± SD)**	21.78 ± 2.72	21.47 ± 2.47	22.05 ± 2.91	0.033
**Gender**				
Male	182 (45.2)	90 (47.9)	92 (42.8)	0.306
Female	221 (54.8)	98 (52.1)	123 (57.2)	
**Marital status**				
Single	377 (93.5)	182 (96.8)	195 (90.7)	0.002
Married	19 (4.7)	2 (1.1)	17 (7.9)	
Divorced/widow	7 (1.7)	4 (2.1)	3 (1.4)	
**Family income (Saudi Riyals)**				
<5000	123 (30.5)	59 (31.4)	64 (29.8)	0.802
5000–10,000	94 (23.3)	44 (23.4)	50 (23.3)	
10,000–15,000	70 (17.4)	36 (19.1)	34 (15.8)	
15,000–20,000	47 (11.7)	20 (10.6)	27 (12.6)	
>20,000	69 (17.1)	29 (15.4)	40 (18.6)	
**Residence**				
Living with family	365 (90.6)	171 (91.0)	194 (90.2)	0.804
Living independently	38 (9.4)	17 (9.0)	21 (9.8)	
**Fast food intake**				
Consumer	351 (87.1)	162 (86.2)	189 (87.9)	0.604
Non-consumer	52 (12.9)	26 (13.8)	26 (12.1)	
Soft drink intake				
Consumer	286 (71.0)	135 (71.8)	151 (70.2)	0.728
Non-consumer	117 (29.0)	53 (28.2)	64 (29.8)	
**Fruit and vegetable intake**				
Yes	134 (33.3)	57 (30.3)	77 (35.8)	0.243
No	269 (66.7)	131 (69.7)	138 (64.2)	
**Physical activity**				
Inactive	144 (35.7)	73 (38.8)	71 (33.0)	0.225
Active	259 (64.3)	115 (61.2)	144 (67.0)	
Smoking				
Yes	80 (19.9)	42 (22.3)	38 (17.7)	0.241
No	323 (80.1)	146 (77.7)	177 (82.3)	
**Daily screen time usage (television, videos, internet, social media) on weekdays**				
I don’t watch	11 (2.7)	7 (3.7)	4 (1.9)	0.480
1–3 h	50 (12.4)	24 (12.8)	26 (12.1)	
less 4–6 h	190 (47.1)	92 (48.9)	98 (45.6)	
>6 h	152 (37.7)	65 (34.6)	87 (40.5)	
**Hours of sleep per night during the week**				
<5 h	68 (16.9)	31 (16.5)	37 (17.2)	0.516
5–8 h	245 (60.8)	110 (58.5)	135 (62.8)	
9–10 h	74 (18.4)	37 (19.7)	37 (17.2)	
>10 h	16 (4.0)	10 (5.3)	6 (2.8)	

(1) Low knowledge (0–8); high knowledge (9–16). (2) *p*-values for continuous variables were estimated using unadjusted linear regression with the knowledge category (low vs. high). *p*-values for categorical variables were estimated using Pearson’s χ^2^ test.

**Table 3:** Adjusted analyses of the consumption of ultra-processed food and the knowledge of ultra-processed food (*n* = 403).

	Knowledge of Food Processing Classification (1)		
UPF consumption	**B (95% CI)**	**SE**	***p*** **-Value (4)**
**Crude model**	−0.258 (−0.428, −0.089)	0.086	0.003
**Model 1 (2)**	−0.257 (−0.428, −0.087)	0.087	0.003
**Model 2 (3)**	−0.241 (−0.414, −0.068)	0.088	0.006

Abbreviations: B, unstandardized regression coefficient; CI, confidence interval. Adjusted R^2^: crude model = 0.019; Model 1 = 0.019; Model 2 = 0.036. (1) Knowledge of food processing classification was analyzed as a continuous score ranging from 0 to 16. (2) Model 1 was adjusted for demographic information (age, sex, marital status, living arrangements, and income level). (3) Model 2 was adjusted for Model 1, physical activity, smoking, sleep duration, and fruit and vegetable consumption. (4) *p*-values were calculated using linear regression of the total frequency of intake of ultra-processed foods.

**Table 4:** Distribution of ultra-processed food patterns according to the participants’ knowledge of food processing classification (*n* = 190).

	Total (*n* = 190)	Low Knowledge (*n* = 96) (1)	High Knowledge (*n* = 94)
	Mean ± SE	Mean ± SE	Mean ± SE
**Bread and bakery products (2)**	162.83 ± 11.33	178.27 ± 16.94	147.06 ± 14.93
**Breakfast cereals (3)**	13.30 ± 3.34	18.26 ± 5.85	8.24 ± 3.11
**Snacks and savory items (4)**	63.54 ± 13.74	69.12 ± 17.17	57.84 ± 21.62
**Dairy products (5)**	86.34 ± 6.95	90.46 ± 10.14	82.14 ± 9.53
**Ready sauces (6)**	22.31 ± 3.53	21.28 ± 5.49	23.37 ± 4.45
**Ready-to-eat or ready-to-heat meals (7)**	154.22 ± 13.17	162.77 ± 20.05	145.50 ± 17.09
**Processed meat, condiments and seafood**	88.47 ± 10.86	91.29 ± 15.99	85.59 ± 14.76

(1) Low knowledge (0–8); high knowledge (9–16). (2) Croissants, white French sticks, toast, fortified bread, peshwari, hamburger buns, cake, doughnuts, toasted mini bread, biscuits, and pastries. (3) Fortified cornflakes, oats, and granola. (4) Industrial chips and packaged salty snacks. (5) Dairy products included processed cheese, cream and ice cream, and yogurt. (6) Ketchup, mayo, mustard, restaurant sauces, and salad dressing. (7) Packaged pre-prepared meals, canned soups and beans, French fries (frozen), and noodles.

**Table 5:** Consumption of nutrients considering the participants’ knowledge of food processing classification (*n* = 190).

Nutrients	Total (*n* = 190)	Low Knowledge (1) (*n* = 96)	High Knowledge (*n* = 94)	Adjusted B (95% CI)	*p*-Value (2)
	Mean ± SD	Mean ± SD	Mean ± SD		
**Carbohydrate (%)**	49.57 ± 7.47	49.82 ± 7.37	49.31 ± 7.59	−0.637 (−2.795, 1.521)	0.561
**Protein (%)**	17.04 ± 5.45	16.64 ± 5.30	17.45 ± 5.60	0.865 (−0.710, 2.441)	0.280
**Fat (%)**	36.28 ± 6.31	36.73 ± 6.62	35.83 ± 5.98	−0.424 (−2.249, 1.401)	0.647
**Trans fat (g)**	1.17 ± 0.80	1.21 ± 0.83	1.12 ± 0.77	−0.076 (−0.307, 0.156)	0.521
**Saturated fat (g)**	25.86 ± 12.10	26.83 ± 12.32	24.86 ± 11.84	−1.641 (−5.112, 1.830)	0.352
**Fiber (g/day)**	16.00 ± 6.93	15.51 ± 6.29	16.50 ± 7.54	1.100 (−0.901, 3.100)	0.280

(1) Low knowledge (0–8); high knowledge (9–16). (2) *p*-values were estimated using linear regression models adjusted for age with the knowledge category (low vs. high).

## Data Availability

The original contributions presented in this study are included in the article/Supplementary Materials. Further inquiries can be directed to the corresponding author.

## Supplementary Materials

The following supporting information can be downloaded at: https://www.mdpi.com/article/10.3390/foods15172955/s1, Table S1. Baseline characteristics of 24-h dietary recall participants and non-participants.

## Abbreviations

The following abbreviations are used in this manuscript:

FoPL	Front-of-package labeling
NOVA	NOVA food classification system
UPF	Ultra-processed food
