# Supplementary material for: Recognition of Food Processing and Its Association with Consumption Patterns and Nutrient Intake Among Saudi University Students
Source: Foods. 2026 Aug 22;15(17):2955. doi: 10.3390/foods15172955 (PMC13564625; doi:10.3390/foods15172955)
Supplement: Supplementary file 1 [file foods-15-02955-s001.zip › foods-4467921-supplementary.pdf]

Table S1. Baseline characteristics of 24-h dietary recall participants and non-participants.

Characteristic	24-h recall participants (n = 190)	Non-participants (n = 213)	P-value (1)
**Age group, n (%)**	20.98 ± 2.376	21.40 ± 2.357	0.076
**Gender, n (%)**			0.100
Male	94 (49.5%)	88 (41.3%)	
Female	96 (50.5%)	125 (58.7%)	
**Marital status, n (%)**			0.002
Single	184 (96.8%)	193 (90.6%)	
Married	2 (1.1%)	17 (8.0%)	
Divorced/widowed	4 (2.1%)	3 (1.4%)	
**Family income, n (%)**			0.057
<5,000 Riyals	51 (26.8%)	72 (33.8%)	
5,000–<10,000	45 (23.7%)	48 (22.5%)	
10,000–<15,000	29 (15.3%)	42 (19.7%)	
15,000–<20,000	22 (11.6%)	25 (11.7%)	
≥20,000	43 (22.6%)	26 (12.2%)	
**Living arrangement, n (%)**			0.896
Living with family	172 (90.5%)	192 (90.1%)	
Living alone	18 (9.5%)	21 (9.9%)	
**Knowledge category, n (%)**			0.141
Low knowledge	96 (50.5%)	92 (43.2%)	
High knowledge	94 (49.5%)	121 (56.8%)	

(1) P-values were calculated using the independent-samples t-test for continuous variables, Pearson's χ^2 test for categorical variables, and Fisher's exact test.
